# Can Motor Arrests in Other Effectors Be Used as Valid Markers of Freezing of Gait?

**DOI:** 10.3389/fnhum.2021.808734

**Published:** 2021-12-16

**Authors:** Nicholas D'Cruz, Alice Nieuwboer

**Affiliations:** KU Leuven, Department of Rehabilitation Sciences, Neurorehabilitation Research Group, Leuven, Belgium

**Keywords:** Parkinson's disease, freezing of gait, motor blocks, conversion, biomarkers

## Background

People with Parkinson's disease (PD) have an increased risk of falling, which is often associated with the manifestation of freezing of gait (FOG) (Pelicioni et al., 2019). Not surprisingly, turning and gait initiation are frequent triggers of FOG as these complex maneuvers require precise control of the center of mass as well as adaptation of the locomotion pattern (Bekkers et al., 2018). Key to the motor deficits of PD is the loss of motor automaticity, defined as the ability to perform movements without attention directed toward the details of movement (Wu et al., 2015). As such, fine-tuning of gait control becomes especially compromised in daily life when locomotion is less regulated by conscious processing in PD. FOG is more imminent when people with PD are multi-tasking and coping with doorways and obstacles (Beck et al., 2015; Mancini et al., 2018). Equally, FOG is more likely when under stress of FOG-anticipation at “freezing hotspots” or when experiencing fear of falling (Economou et al., 2021). While recognizing that there may be common-end mechanisms between FOG, dynamic balance disturbances, attention and anxiety, in this view point we want to focus on the relevance of studying freezing of repetitive movements of the extremities as a handle on understanding FOG.

The main bottleneck to better understand when and why FOG emerges and how to manage it is the lack of valid markers of FOG, justifying the search for models of freezing in other effectors than in gait. Several instrumented methods for measuring FOG episodes in daily life as well as during standardized lab tests are currently in the validation pipeline (Mancini et al., 2021; Pardoel et al., 2021). However, as yet, they have not demonstrated robust construct and predictive validity, particularly for short and more subtle episodes that are likely to occur in early disease and when ON-medication (Mancini et al., 2019). Digitized outcome measures of FOG vary from fairly simple detection algorithms, as derived from wearable sensor signals, to artificial intelligence-based methodologies (Pardoel et al., 2021). Most of these algorithms are apt in capturing the high frequency movement phenomena associated with FOG, including leg trembling or small shuffling steps (Mancini et al., 2021; Pardoel et al., 2021). Yet, “akinetic FOG,” displaying no discernable movement during the episode is more difficult to distinguish from voluntary stops (Cockx et al., 2021). Also, the variable and often interrupted gait bouts observed in daily life provide a noisy background from which to pick up FOG-signals, creating high rates of false positives (Mazilu et al., 2015). The heterogeneous clinical manifestation of FOG by itself also complicates validation work as it affects the robustness of the gold standard measure of FOG. At present, the percentage time frozen (%timeFR) determined during expert video annotation of standardized gait tests constitutes the best reference test, most notably when performing turning tasks (Morris et al., 2012). However, turning is also a hazardous test when no supervision is available to prevent falling, especially in a home setting. As such, markers of freezing which are safe, reliable, responsive and predictive of FOG along the disease progression axis are much needed.

## State of the Art on Freezing in Other Effectors

Our group was one of the first to acknowledge the remarkable similarity between features of FOG and motor arrests when performing sequential finger and writing movements uni- and bimanually (Nieuwboer et al., 2009; Vercruysse et al., 2012). While freezing was worse in bimanual sequences, it also occurred in uni-manual ones, suggesting that bilateral co-ordination was a contributing but not a deciding factor for triggering a freezing response (Vercruysse et al., 2012). Both types of freezing were typically preceded by the so-called “sequence effect,” defined as the rapid diminishment of amplitude and/or speed with each repetition (Tinaz et al., 2016). Interestingly, we found that motor arrests arising from the sequence effect were triggered by bringing the motor system in overdrive at two dimensions, i.e., by reducing the scale as well as by increasing the rhythm of movement cycles (Nieuwboer et al., 2009; Vercruysse et al., 2012). The pathophysiology of the sequence effect can be understood as a failure of central motor energy, which is partly responsive to levodopa (Tinaz et al., 2016). Indeed, magnetic resonance imaging (MRI) showed that levodopa restored the function of the motor circuit associated with better writing sizes, as performed in the scanner, but did not alter “progressive micrographia” (Wu et al., 2016). Interestingly, we also found “sequence effect-like” abnormalities during accelerated weight-shifting sequences without stepping in a standing-in-place task (Dijkstra et al., 2021). Here, the axial amplitudes of weight-shifts were reduced and showed earlier breakdown in freezers compared to non-freezers and this more so in OFF compared to ON medication (Dijkstra et al., 2021). Returning to non-gait freezing, not only impaired regulation of motor vigor, but also increased energy in the high frequency bands appeared to be involved in sequence breakdown, resembling the oscillatory features of FOG (Vercruysse et al., 2012). These dysrhythmic abnormalities were interpreted to indicate faulty initiation-termination responses (Stegemöller et al., 2017), or arising from a pathological frequency content of the antagonistic muscles, albeit distinct from resting or action tremor frequencies (Scholten et al., 2016).

As upper limb freezing was brought on when people with PD were subjected to similar motor challenges as in FOG, we recently investigated whether producing up-and-down strokes on a writing tablet within a funnel figure, with wide, narrow and transitioning pieces, elicited motor arrests similar to presenting a doorway to trigger FOG in a gait lab or in the home (Heremans et al., 2019). We found that motor arrests were most prominent in the narrow and decreasing parts of the funnel, despite the fact that this motor adaptation task provided target lines, expected to energize and provide feedback on the scale movement. Similar to earlier findings, the frequency and duration of the motor arrests more than doubled when motor load was increased by imposing fast speed conditions and this while subjects were “ON” medication.

## Construct Validity of Freezing in Other Effectors

As for construct validity of non-gait freezing, a review on freezing episodes in a variety of tasks, i.e., handwriting, hand and foot tapping and speech revealed that the clinical manifestation of these events appeared to be overlapping (Vercruysse et al., 2014a). However, a profound definition of what exactly constitutes a non-gait freezing event is still lacking, especially with regards to including hastening epochs and the transition phase between normal movement and freezing. So far, pragmatic definitions were employed largely based on visual criteria for rating FOG (Vercruysse et al., 2012; Heremans et al., 2019). Also, in 20 out of the 23 studies of the above-mentioned review in which the relationship between freezing in other effectors and FOG was explored, non-gait freezing was more prevalent in patients with FOG or correlated with higher FOG-severity. However, none of these studies applied formal classification statistics to discern whether non-gait freezing can accurately distinguish between groups with and without FOG.

As for “fast funnel freezing,” freezing events occurred in 23 out of 49 patients and its frequency was correlated to self-reported FOG severity, though this correlation was not found for %timeFR during the funnel task (Heremans et al., 2019). As well, a substantial number of people without FOG had motor arrests in the funnels. The opposite pattern was also reported, namely that out of 16 people with FOG only 9 displayed upper limb freezing (Scholten et al., 2016). All this could suggest three things. First, upper limb freezing is less indicative of FOG than suggested previously, questioning its value as a proxy marker. Second, the most optimal method to elicit freezing in other effectors (high speed conditions) was not always employed, which may have precluded the events from occurring. Third, it could be that people without FOG but with freezing in other body parts have a higher likelihood to convert to FOG showing the potential for repetitive movement paradigms to serve as predictive markers for FOG.

## Predictive Validity of Freezing in Other Effectors

**Figure 1:**
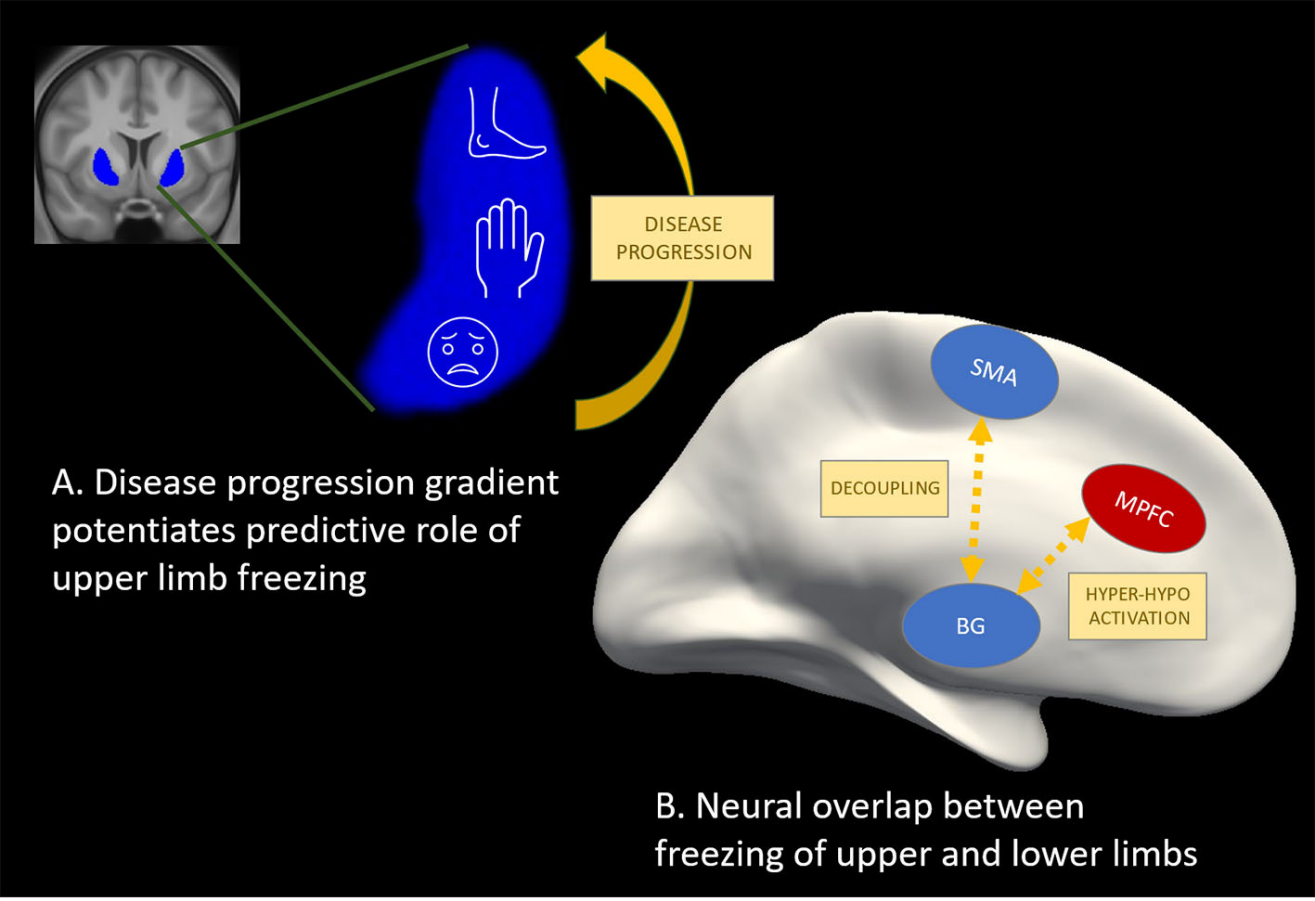
Potential for other effectors as valid markers of freezing of gait. **(A)** The motor representation in the putamen (Nambu, 2011) (in blue) aligns with the gradient of dopaminergic loss in the putamen (Kish et al., 1988) (from caudal to rostral) such that the face and upper limbs are affecter earlier, potentiating predictive utility of upper limb freezing. **(B)** Hyper-activation (in red) and hypo-activation (in blue) in cortical and sub-cortical regions as well as desynchronization or decoupling between these regions has been shown during freezing episodes in finger tapping (Vercruysse et al., 2014b; Brugger et al., 2020), foot pedaling (Shine et al., 2013a,b; Matar et al., 2019), and during real gait (Pozzi et al., 2019) studies. These findings point to a common neural mechanism underlying freezing in gait and non-gait effectors, which is promising for future work aiming to further explore freezing mechanisms as well as therapeutic effects on the freezing circuitry.

Prospective study conducted by Delval et al. demonstrated, that episodic events during foot-tapping, hand-tapping, and syllable repetition in early-stage PD patients without FOG were predictive of FOG emerging in the next two years, albeit in a small cohort of 30 subjects (Delval et al., 2016). Notably, the speed of the alternating tapping and speech, tasks that were objectively measured, were imposed by a metronome with increasing rhythms eliciting freezing as well as hastening events. Recently, we also conducted a prospective study on 60 patients without FOG to assess the predictive value of several motor and non-motor outcomes as markers of FOG conversion (D'Cruz et al., 2020). Over a follow-up of two years, 20% of patients converted. Next, we investigated the contributions of amplitude, rhythm, coordination and the freezing ratio exhibited during repetitive motor tests in the extremities as well as during gait and turning. Unlike in Delval et al., movement tests were largely self-generated and mostly delivered at a comfortable pace. After applying robust techniques to reduce the number of variables, two main components in a multivariable model were found to predict FOG conversion within the next year with an area under the curve of 0.79. The two main components were: (1) worse disease severity (on a number of specific items including upper limb tasks) and (2) worse finger tapping movements (smaller amplitude, inconsistent timing and poor coordination). While these results suggested that altered movement generation during repetitive movements is central to FOG, it is possible that disease progression was also inadvertently captured by the deterioration of the quality of repetitive movements. Recently, it was shown that a digitized alternating finger tapping task was the most sensitive and specific motor test for detecting conversion to PD prospectively in a prodromal cohort with idiopathic REM sleep disorder (Fereshtehnejad et al., 2019). Furthermore, Figure 1A illustrates that the motor representation in the putamen aligns with the gradient of dopaminergic loss in the putamen (from caudal to rostral) such that the face and upper limbs are affecter earlier (Kish et al., 1988; Nambu, 2011), potentiating the role of the degradation of upper limb motion for predicting the onset of FOG.

## Neural Correlates of FOG and Freezing in Different Effectors

Mobile neuroimaging techniques as well as local field potential recordings are increasingly applied to better understand the brain circuit dysfunctions underlying actual FOG episodes obtained during over-ground walking (Tard et al., 2015; Pozzi et al., 2019). While such methods are developed further, a number of studies have used motor arrests provoked during repetitive foot or finger motion to study the neural mechanisms related to freezing events with non-mobile electroencephalography set-ups (EEG) or in a MRI scanner. Functional MRI and EEG mainly highlight cortical activations and their outcomes are highly task-specific, limiting the interpretation of these findings to a heterogeneous phenomenon such as freezing. Taking these drawbacks in consideration, the most influential model of FOG (Lewis and Shine, 2016) stems from a “foot pedaling” fMRI-paradigm executed while lying in a scanner and while “moving forward” through a virtual reality (VR) corridor. When confronted with conditions of high cognitive load in the VR, episodes of increased pedaling latency were found to be associated with decreased activation in sensorimotor cortical and several basal ganglia regions (Shine et al., 2013a). In contrast, frontoparietal activation was higher compared to successful pedaling, suggesting that cortico-subcortical decoupling underlies freezing events. A strikingly similar cortical-basal ganglia mismatch of hyper and hypo-activity, respectively, during motor blocks of repetitive finger movements was also found (Vercruysse et al., 2014b), suggesting some overlap between the foot and finger studies (Vercruysse et al., 2014a) as displayed schematically in Figure 1B. However, differences were apparent too with respect to the involvement of the superior structures of the brainstem known to control gait and posture, which only came out of the pedaling study. When showing narrow and not wide doorways during the foot pedaling task, freezing events were accompanied with hypo-activity in pre-Supplementary Motor Area (pSMA) and reduced connectivity between the pSMA and the Subthalamic Nucleus, suggesting involvement of the hyperdirect pathway (Matar et al., 2019). As well, the cortico-subcortical decoupling was already noticeable in the run-up to freezing episodes of the feet (Matar et al., 2019), similar to FOG (Pozzi et al., 2019). All this work has substantially influenced current thinking on FOG as a phenomenon which can be brought on by various failures in different task-related networks, converging toward a common neural pathway dysfunction (Lewis and Shine, 2016).

As for seated EEG, one recent study demonstrated that movement initiation of a finger sequencing task displayed reduced beta-desynchronization in the SMA and this more so in freezers compared to non-freezers (Brugger et al., 2020). Interestingly, the SMA was found to be a central hub in the locomotor fine-tuning network in young healthy people while experiencing gait perturbations as highlighted by PET-imaging of the brain's glucose metabolism (Hinton et al., 2019). In line, the SMA proved to be less involved when people with FOG were undergoing a FOG-provoking gait-protocol compared to those without FOG, assessed with PET (Tard et al., 2015). A second EEG study showed that an increase of left prefrontal beta band synchronization was predictive of upper limb freezing, pointing to the relevance of prefrontal executive dysfunction in analogy to FOG (Scholten et al., 2020). Taken together, it seems that non-gait freezing paradigms are able to capture components of the supraspinal locomotor networks and how it is disrupted during freezing.

## Conclusion and Future Direction

We have highlighted that studying freezing in other effectors has great potential as a model for investigating component-mechanisms of FOG. However, we also showed that further validation of non-gait freezing as a behavioral biomarker of FOG is indicated. Therefore, prospective cohort studies are needed including recently diagnosed patients with PD, as well as positive control groups with FOG to be able to track progression of both gait and non-gait freezing-severity. As for measuring repetitive finger movements, keyboards as well as tablets and smartphones technology could be used to quantify motor blocks (Trager et al., 2020) and foot tapping assessments can be quantified by wearable sensors at the feet and ankles (Rovini et al., 2017). These research paradigms are relatively easy to apply in a home setting in a sitting position with remotely controlled reminders or as part of telemedicine platforms. In a lab environment, these tests can safely be combined with sensitive tests of FOG, such as performing 360° turns. As highlighted in this view point, stringent conditions to bring subjects to the limits of their performance need to be employed so that longitudinal change in symptom progression can be captured and more importantly so that freezing events actually come to the fore. To move the field forward, we further recommend to clarify and refine the clinical definition of non-gait freezing events, in analogy to FOG, to serve as the gold standard criterion for future automatic detection.

## Author Contributions

ND drafting manuscript. AN conceptualizing and revising manuscript. All authors contributed to the article and approved the submitted version.

## Conflict of Interest

The authors declare that the research was conducted in the absence of any commercial or financial relationships that could be construed as a potential conflict of interest.

## Publisher's Note

All claims expressed in this article are solely those of the authors and do not necessarily represent those of their affiliated organizations, or those of the publisher, the editors and the reviewers. Any product that may be evaluated in this article, or claim that may be made by its manufacturer, is not guaranteed or endorsed by the publisher.
